# Inoculation of Tubercle

**Published:** 1870-03

**Authors:** 


### Inoculation of Tubercle.

The subject of the inoculation of tubercle has been examined carefully by Cohneim and Frankel. Their experiments were made with guinea-pigs, and they found that the introduction of tubercle from the human subject was followed, if the animal survived long enough, by the development of unmistakable miliary tubercle in the inoculated animal; results which agree with what may be called the positive experiments of Villemin.

They also found, however, that the same result—namely, the development of miliary tubercle—followed the introduction of other substances than tubercle.

Portions of apparently healthy organs, pieces of cancerous tumours, condylomata, sarcomata, pieces of blotting paper, charpie, vulcanised caoutchonc or gutta percha, when introduced into the peritoneal cavity, gave rise to a uniform result, namely, the development of tubercle in the animal subjected to the operation. These results are directly contradictory of Villemin's negetive experiments, but agree with those of Dr. Sanderson and Dr. Wilson Fox, noticed in the number of the Journal for August, 1868.

The introduction of any of the substances which were employed was followed by peritonitis, and about one-half of the animals died from this cause in from one to four days after the operation—of course at too early a period to afford any evidence of tuberculosis. In those who survived, the peritonitis became circumscribed, and a tumour with a capsular envelope was formed. Within the capsule the foreign body was to be found, when charpie, caoutchoue, or similar substances had been used, but in no instanc was it possible to recognize a trace of any of the portions of the organism which had been introduced, whether normal or pathological. The alteration which the portions of cancer, tubercle, &c., underwent, were so complete as to entirely prevent their recognition, even with the microscope. It is evident, according to the authors, since the systemic infection cannot be the direct result of the inoculation—a fact which is obvious in cases where caoutchouc, etc., have been introduced, and which is in the highest degree probable as regards the introduction of animal substances—that the cause of the phenomena must be sought elsewhere. The really effective agent is the dead and generated pus which is found, in every instance, as the result of the reactive inflamation after the introduction of the foreign bodies. This pus is the true foreign body, and its absorption into the economy is the cause of the subsequent pathological changes.—*Virchow's Archiv.—Dublin Quarterly Journal.—Braithwaite.*

